# Adverse childhood experiences and chronic pain among children and adolescents in the United States

**DOI:** 10.1097/PR9.0000000000000839

**Published:** 2020-08-13

**Authors:** Cornelius B. Groenewald, Caitlin B. Murray, Tonya M. Palermo

**Affiliations:** a Center for Child Health, Behavior and Development, Seattle Children's Research Institute, Seattle, WA, USA; b Department of Anesthesiology and Pain Medicine, University of Washington, Seattle, WA, USA

**Keywords:** Adverse childhood experiences, Childhood, Adolescence

## Abstract

**Objective:**

To evaluate the association between adverse childhood experiences (ACEs) and chronic pain during childhood and adolescence.

**Methods:**

Cross-sectional analysis of the 2016-2017 National Survey of Children's Health, including 48,567 child participants of 6 to 17 years of age. Parents of children reported on 9 ACEs. Chronic pain was defined as parents reporting that their children had *“frequent or chronic difficulty with repeated or chronic physical pain, including headache or other back or body pain during the past 12 months.”* Multivariate logistic regression analysis adjusted for sociodemographic and health-related factors.

**Results:**

In this nationally representative sample, 49.8% of children were exposed to one or more ACEs during their lifetime. Children with exposure to 1 or more ACEs had higher rates of chronic pain (8.7%) as compared to those with no reported ACEs (4.8%). In multivariate analysis, children with ACEs had increased odds for chronic pain (adjusted odds ratio [aOR]: 1.6, 95% confidence interval [CI]: 1.3–2.2, for 0 vs 1 ACE and aOR: 2.7, 95% CI: 2.1–3.4 for 0 vs 4+ ACEs). The strongest associations of individually measured ACEs with chronic pain included financial instability (aOR: 1.9, 95% CI: 1.6–2.2), living with a mentally ill adult (aOR: 1.8, 95% CI: 1.5–2.2), and having experienced discrimination based on race (aOR: 1.7, 95% CI: 1.3–2.2).

**Conclusions:**

Children and adolescents with ACEs had increased risk for chronic pain, and this association increased in a dose-dependent fashion.

## Abstract 2

In this large, nationally representative study, children exposed to adverse childhood events had increased rates of chronic pain (8.7%) as compared to their peers (4.8%).

## 1. Introduction

Adverse childhood experiences (ACEs) are defined as exposure to potentially traumatic experiences before 18 years of age that could have long-lasting effects on health and well-being.^3^ ACEs include events that the child experiences or witnesses (eg, serious injury/accident, parent divorce, witnessing violence), physical abuse (being assaulted), sexual abuse, or emotional abuse/neglect (eg, being ridiculed/ignored by parents) as well as events that undermine a sense of safety such as growing up in a household with substance abuse, mental health problems, or an incarcerated parent. Before 18 years of age, approximately 45% of children in the United States experience ACEs, the most common of which include economic hardship (22.5%) and parental divorce (21.9%).^4,22^ Exposure to ACEs, in some cases, leads to toxic stress activation, which disrupts normal brain development^9^ and increases risk of physical (eg, cardiovascular disease and obesity) and mental health disorders (eg, substance abuse and depression) across the lifespan.^7^ Furthermore, ACEs often co-occur and have a dose-dependent effect, such that exposure to multiple ACEs further increases the risk for mental health disease and substance abuse.^12^

Nelson et al. recently introduced a novel conceptual framework for understanding the effects of ACEs on the development of childhood chronic pain, based on the biopsychosocial model of pain.^16^ According to this model, chronic pain may develop from the interaction of persistent biological, psychological, and social stressors experienced during childhood. Understanding the impact of ACEs on chronic pain is particularly relevant, given that up to 25% of children in the United States report having chronic pain.^13^ However, few studies have examined the prevalence of ACEs and their relationship to chronic pain among children in the United States. Indeed, research to date has focused on a few specific clinical pain samples including fibromyalgia,^15^ abdominal pain,^11^ complex regional pain syndrome,^28^ and migraine headache^8^ and those presenting for multidisciplinary chronic pain management.^18^ Taken together, the evidence available suggests that youth with chronic pain conditions report ACEs more frequently than youth without chronic pain. However, there are no data concerning the association between ACEs and chronic pain in large, nationally representative samples of youth in the United States.

This is an important gap in knowledge because there is increasing evidence that the impact of ACEs on childhood health outcomes in the United States may be improved by family-based interventions including parent education, mental health counseling, and home health visits.^14^ Better understanding of patterns and predictors of ACEs among children with chronic pain may allow clinicians and healthcare policymakers to develop and implement policies and programs aimed at improving health outcomes in our large population of children with chronic pain.

Therefore, the aims of this study were to determine the epidemiological associations between ACEs and childhood chronic pain in the United States. We hypothesize that exposure to ACEs would be associated with increased likelihood of having chronic pain, even after controlling for sociodemographic and health characteristics. In addition, we also hypothesize that cumulative exposure to multiple ACEs would be associated with an increased likelihood of chronic pain (as compared to exposure to 1 ACE).

## 2. Methods

This study was a cross-sectional analysis of the 2016 and 2017 National Survey of Children's Health (NSCH).^11^ National Survey of Children's Health was funded by the Health Resources and Services Administration's Maternal and Child Health Bureau, an agency of the U.S. Department of Health and Human Services. The survey was then conducted by the Center for Disease Control and Prevention. National Survey of Children's Health has been conducted since 2011/2012 with the primary purpose to measure childhood physical and mental health and associated determinants. In 2016 and 2017, NSCH for the first time included a measure of childhood chronic pain, which provides an opportunity to measure the epidemiological association between ACEs and childhood chronic pain in a large nationally representative sample. All data were parent-reported. Of the 51,156 children, 6 to 17 years of age, captured in the 2016-2017 NSCH, we excluded 2589 (5%) with missing data on one of the variables of interest, leaving a final sample of 48,567 for analysis. Seattle Children's Hospital institutional review board determined this study to be exempt from review because all data from NSCH are publicly available and nonidentifiable.

## 3. Measures

### 3.1. Chronic pain

Parents were asked, “During the past 12 months, did this child have frequent or chronic difficulty with repeated or chronic physical pain, including headaches or other back or body pain?” Presence of chronic pain was coded by a “yes” response to this item. The specific duration or severity of chronic pain was not available for analysis.

### 3.2. Adverse childhood experiences

The primary predictor variables were 9 questions about ACEs over the child's lifetime that were reported by the parent. Five of the survey items, including: (1) divorce or separation of parent; (2) parent served time in jail; (3) child witnessed domestic violence; (4) lived with someone who was mentally ill or suicidal; and (5) lived with someone with substance abuse problems, were adopted from the Behavioral Risk Factor Surveillance System ACE Module (Centers for Disease Control and Prevention). Additional survey items, including: (1) treated or judged unfairly due to race/ethnicity; (2) experienced death of parent; (3) child was a victim or witness of neighborhood violence; and (4) child suffered hardship due to low family income, were developed based on input from a technical expert panel involved with the survey to capture potentially stressful life-course events and experiences. Some of the survey items used a Likert scale and responses categorized as “somewhat often” or “very often” were coded as a positive (yes) response and “rarely” or “never” as a negative (no) response. All ACEs were categorized as binary (Y/N) and also summed to calculate a composite score reflecting the number of ACEs each participant experienced.

#### 3.3. Covariates

Data were collected on the following sociodemographic variables: age, sex (male vs female), race/ethnicity (White, non-Hispanic, Black, non-Hispanic, Hispanic, and Other or multiracial), household income (<100% federal poverty level [FPL], 100%–199% FPL, 200%–399% FPL, 400% or greater FPL), insurance type (private, public only, uninsured), and primary language spoken at home (English vs non-English). We also controlled for parent-reported child mental health conditions (depression and anxiety) and overall health (rated by parent as excellent, very good, or good vs fair or poor).

#### 3.4. Data analysis plan

Analyses were conducted with Stata version 14.2 (StataCorp, College Station, TX).^23^ Statistical tests were 2-sided with α level set at 0.05. We adjusted for the complex survey design of NSCH using sampling weights, stratification, and clustering. Thus, our estimates are nationally representative of the noninstitutionalized childhood population in the United States.

To address the primary aim, we first estimated rates of ACEs in the sample and then determined whether children with 1 or more ACEs had increased rates of chronic pain. Differences in unadjusted rates of chronic pain based on number of ACEs were determined using χ^2^ analyses. We then performed multivariate logistic regression to determine the association between ACEs (having any ACEs) and chronic pain, adjusted for sex, race, ethnicity, poverty level, insurance coverage, language spoken at home, general health, anxiety, and depression.

Next, we determined the prevalence of individual ACEs among children with chronic pain as compared to children without chronic pain using χ^2^ analysis. Finally, we then performed multivariate logistic regression to analyze the association between each individual ACE and chronic pain, adjusted for sex, race, ethnicity, poverty level, insurance coverage, parent education level, language spoken at home, general health, anxiety, and depression.

## 4. Results

### 4.1. Sample characteristics

Our sample included 48,567 participants weighted to represent 46.5 million children of 6 to 17 years of age nationally. Characteristics of the childhood sample are displayed in Table 1 and are representative of the U.S. childhood population. Among all participants, parents of 50.2% of children reported that their child had never experienced an ACE, whereas 49.8% were exposed to one or more ACEs. The prevalence of parent-reported chronic pain among all participants was 8.2%, whereas 8.4% had anxiety and 3.7% had depression.

**Table 1:**
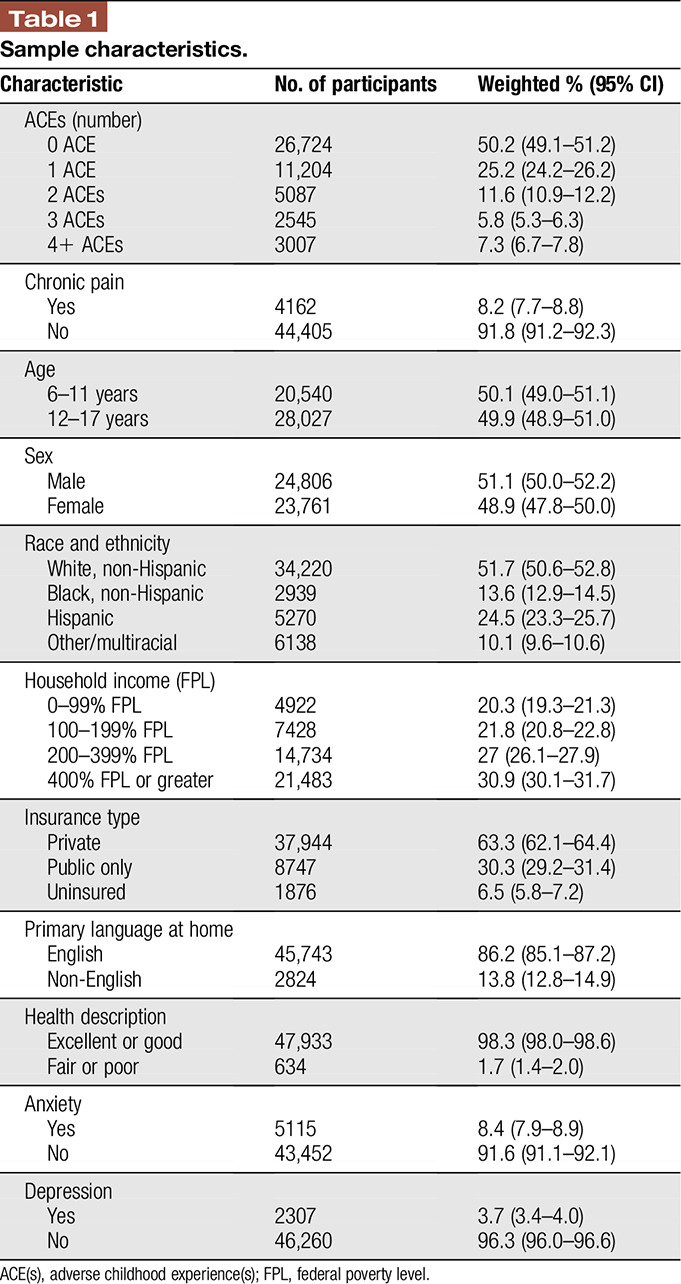
Sample characteristics.

### 4.2. Association between ACEs and chronic pain

The prevalence of chronic pain varied according to exposure to ACEs: Among children with no ACEs, the prevalence of chronic pain was 4.8%, as compared to 8.7% among children who experienced at least one ACE. The prevalence of chronic pain increased to 18.4% among children who experienced 4 or more ACEs (Table 2). These associations held in multivariate logistic regression analysis: children with 1 ACEs vs 0 ACE had 60% increased odds of chronic pain (adjusted odds ratio [aOR] 1.6, 95% confidence interval [CI]: 1.3–2.2), whereas children with 4+ ACEs had 170% increased odds for having chronic pain (aOR: 2.7, 95% CI: 2.1–3.4) vs children with 0 ACEs (Table 3).

**Table 2:**
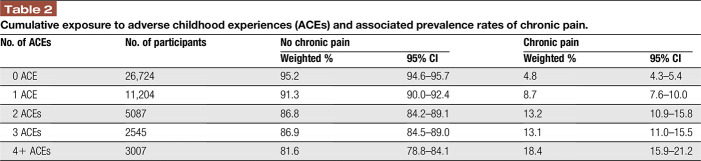
Cumulative exposure to adverse childhood experiences (ACEs) and associated prevalence rates of chronic pain.

**Table 3:**
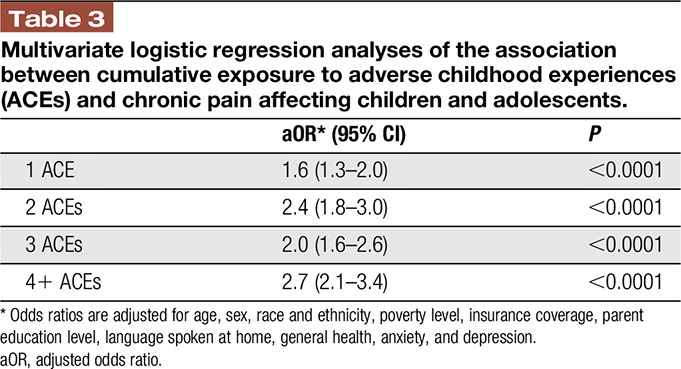
Multivariate logistic regression analyses of the association between cumulative exposure to adverse childhood experiences (ACEs) and chronic pain affecting children and adolescents.

### 4.3. ACEs experienced by children with chronic pain

**Figure 1.**
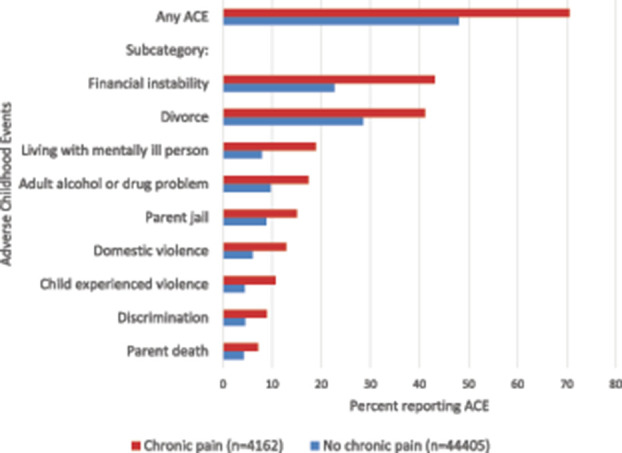
Rates of adverse childhood experiences among children with chronic pain as compared to children without chronic pain.

**Figure 2.**
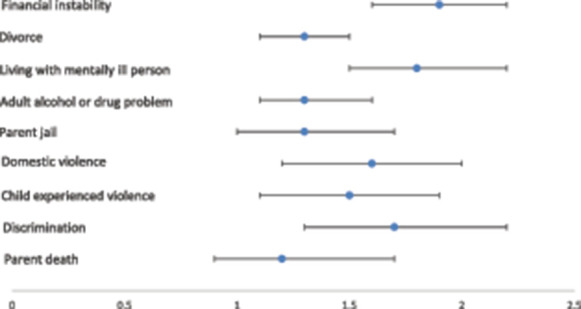
Multivariate associations between individual adverse childhood experiences (ACEs) and chronic pain. Figure presents adjusted odds ratios and 95% confidence intervals for each ACE. *Odds ratios are adjusted for sex, race and ethnicity, poverty level, insurance coverage, parent education level, language spoken at home, general health, anxiety, and depression.

More than 70% of children with chronic pain had experienced at least one ACE during their lifetime as compared to 48% of children without chronic pain (*P* < 0.0001) (Fig. 1).The most common ACEs experienced by children with chronic pain included financial instability (43.1%) and parent divorce (41.1%), which were also the ACEs most commonly experienced by children without chronic pain. The prevalence of each of the individually measured ACE items as measured in the NCHS were higher among children with chronic pain as compared to peers without chronic pain (Fig. 1). After adjusting for sociodemographic and health variables using multivariate logistic regression analysis, associations between 7 of the 9 individual ACEs and chronic pain remained significant (Fig. 2). The strongest associations included that children with chronic pain had increased odds for financial instability (aOR: 1.9, 95% CI 1.6–2.2), living with a mentally ill adult (aOR: 1.8, 95% CI: 1.5–2.2), and having experienced discrimination based on race (aOR: 1.7, 95% CI: 1.3–2.2). In multivariate analysis, children with chronic pain did not have increased odds of experiencing a parent death or parent jail time.

## 5. Discussion

Although there has been great empirical interest in the topic of adverse childhood experiences (ACEs) and risk for chronic pain in adulthood, very few studies have examined ACEs during childhood, the period of life when chronic pain commonly begins. This study is the first to evaluate the association between ACEs and chronic pain in a large, nationally representative sample of over 48,000 children across the pediatric age range (ages 6–17 years). Using data from the 2016 and 2017 National Survey of Children's Health, we found that prevalence of chronic pain varied according to exposure to ACEs, with youth experiencing the most ACEs having the greatest likelihood of chronic pain. Controlling for several demographic and clinical covariates, exposure to one or more ACEs was associated with a 60% to 170% increased likelihood of having chronic pain. As ACE scores increased in the population, so did the rates of chronic pain. Indeed, more than 18% of children with 4+ ACEs had chronic pain. This finding suggests that increased and perhaps chronic exposure to ACEs are key contributing drivers to chronic pain early in life. Our study extends findings in adult samples where childhood adversity predicts future chronic pain^10^ in a dose-dependent relationship.^32^ It also extends findings from small clinical samples of children presenting to tertiary pain centers with specific chronic pain conditions (eg, fibromyalgia and headache). For example, Nelson et al. found that over 80% of children presenting to a multidisciplinary pain clinic reported at least 1 ACE, with 20% reporting lifetime exposure of ≥2 ACEs.^17^

It is important to note that using broad measures of ACEs such as in this study confers a high base rate in the population; in our study, 50% of the population were reported to have experienced ACEs. This may have limited our ability to explain why some youth with ACEs have chronic pain, and others do not. Indeed, most children with ACEs in the study did not have chronic pain. However, our study does suggest that ACEs are associated with chronic pain and may increase risk. The results of this study contribute to the limited evidence base on the association between early childhood adversities and chronic pain in childhood and suggest several promising avenues for future research. It will be critical for future research to identify whether there are sensitive pediatric developmental periods (eg, early childhood vs adolescence) during which the experience of stressful or potentially traumatic events are more likely to confer risk for chronic pain; such knowledge will determine the role of timely interventions to modify developmental mechanisms of chronic pain.

Our results suggest that certain ACEs may be more robustly associated with risk for chronic pain. In particular, children who reported living with a mentally ill or severely depressed person and those experiencing financial instability were more likely to have chronic pain as compared to children experiencing parental divorce or parent death. Thus, ACEs related to instability in the immediate family context may play an important role in the development and maintenance of pediatric pain.^6^ These findings are important, given the abundance of research supporting the influence of parent and family factors on pain and disability in clinical samples of youth with pediatric pain.^20,21^ Moreover, it is well documented that chronic pain tends to cluster in families,^33^ with parents of children with chronic pain also often suffering from chronic pain.^30^ In applying a broader, intergenerational perspective, it is possible that shared exposure to stressful and potentially traumatic family environments, along with its impact on family relationships and parenting, may help to explain the development and maintenance of chronic pain within families.^24^

Nelson, Cunningham, and Kashikar-Zuck (2017) outlined a conceptual framework of the relationship between ACEs and the development of chronic pain in youth. Adapted from the biopsychosocial model of pain, their model highlights biological (ie, allostatic load), psychological (ie, cognitive processes and psychological comorbidities), and social (ie, family and peer/social environment) risk factors as affecting health outcomes including chronic pain and pain-related functional outcomes. This research provides compelling evidence that early life adversities contribute to the development of childhood chronic pain. Although studies support a causal relationship between ACEs and poor health outcomes, underlying mechanisms remain poorly described. As highlighted by Nelson's model, there is emerging evidence suggesting that toxic and chronic stress associated with ACEs may induce epigenetic, hormonal, and immune changes (eg, central sensitization, stress sensitization, HPA activation, and allostatic load) leading to increased risk for chronic pain.^16^ For example, You and Meagher^31^ found that childhood adversity is associated with enhanced central sensitization as measured by larger areas of capsaicin-induced secondary allodynia, which may underlie the link between ACEs and chronic pain development. Furthermore, early life stress may induce epigenetic changes in pain-related gene expression thereby providing a manner for environmental factors to influence chronic pain development.^5^ In addition, several factors may mediate the relationship between ACEs and chronic pain, including sleep disruption^29^ and posttraumatic stress symptoms (PTSS).^1^ Noel et al. found that children with chronic pain had experienced more lifetime stressful events and had elevated PTSS as compared to a matched cohort without chronic pain.^19^ Furthermore, a recent longitudinal study found that PTSS in adolescence mediated the association between childhood maltreatment and adult chronic pain symptoms.^2^ Thus, it is possible that treating the modifiable sequelae of childhood adversities (eg, PTSS) may aid in reducing risk for the development and maintenance of chronic pain.

Overall, these findings highlight the need for routine screening of ACEs in children with chronic pain. Improving education and skills to screen for ACEs among general and specialty care providers may dramatically improve appropriate identification and referrals to psychological treatment for children with chronic pain. Augmenting traditional pain-focused cognitive behavioral therapy with specific trauma-focused cognitive behavioral therapy may also be appropriate depending on presence of PTSS. However, there is a critical need for comprehensive, interdisciplinary research aiming to identify underlying biopsychosocial causal pathways as targets of tailored prevention and intervention. Moreover, ACEs may be a barrier to effective pain management.^18^ For example, families with financial instability may be unable to attend intensive pain rehabilitation programs. Parental separation and parental mental health conditions may also prevent children from getting the medical care they need. Future research should determine whether and how ACEs may lead to poor treatment outcomes among children with chronic pain.

This study has significant strengths, including the use of a nationally representative database, large sample of children with chronic pain, inclusion of a comparison group, and measurement of multiple types of childhood adverse experiences. Despite significant strengths, this study has several limitations that should be acknowledged. First, this is a cross-sectional analysis and the causal relationship between ACEs and chronic pain cannot be determined. It is possible that early life adversities heighten susceptibility for developing chronic pain; at the same time, having a chronic pain problem may also increase vulnerability for an amplified reaction to stressful life adversities or continuation of exposure to ACEs. Prospective research examining ACEs as they relate to the development of chronic pain from childhood to adulthood can provide a clearer picture of how these constructs influence each other over time. In addition, ACEs were reported retrospectively by parents, potentially introducing reporting bias among parents less likely to report sensitive information. However, to minimize bias, participants were told that their answers would be deidentified. Moreover, event-specific information on the severity, frequency, duration, and recency of exposure of each ACE was not assessed. Several ACEs, such as physical and sexual abuse, were not asked about. Although previous studies suggest that ACEs are interrelated, our results may underestimate the true prevalence of ACEs in the U.S. population. Another limitation is that the National Survey of Children's Health includes a limited assessment of chronic pain. As is often the case in large survey data sets, to cover a range of health conditions, chronic pain was only assessed with a single item. Ideally, chronic pain would be assessed using multiple dimensions to be more consistent with contemporary chronic pain definitions, such as the International Association with the Study of Pain definition of chronic pain as pain that persists or recurs for 3 months.^13^ Furthermore, the NSCH captured parent-reported pain, but not child-reported pain, which could introduce bias. Although parents can offer a valid clinical perspective on their child's pain, it is unlikely that there is complete interrater agreement.^27^ Regardless, we believe we were able to conservatively categorize youth in terms of pain chronicity. Among our sample of school-aged children, 8.2% had frequent difficulty with repeated or chronic physical pain problems over the preceding 12 months as reported by their parents. To date, other large studies have shown that 15% to 25% of children report chronic pain but 5% to 8% have disabling chronic pain requiring more intensive intervention. Thus, our sample likely represents children with moderate to severe chronic pain problems. Still, critical dimensions of chronic pain such as the severity, duration, and functional impact of pain were unfortunately not included.

In conclusion, results of this nationally representative study in over 48,000 children and adolescents with and without chronic pain in the United States found that ACEs are strongly associated with chronic pain. Chronic pain providers should ideally screen and identify ACEs in their patients. Although ACEs cannot be undone, psychologists, counselors, and social workers may be able to target interventions to build personal resiliency, address family dysfunction, and teach pain-coping skills to improve health outcomes in at-risk youth. Drawing from biopsychosocial models of pain, future prospective research should focus on deepening knowledge on the underlying mechanisms that may explain the link between ACEs and chronic pain, including modifiable psychological and family-based targets to improve prevention and treatment approaches. In addition, research should focus on the effectiveness of interventions that target chronic pain among children with ACEs.

## Disclosures

The authors have no conflicts of interest to declare.

This work was partially funded by grants from the National Institutes of Health: (grant number K23HL138155 awarded to C.B.G.) and (grant number F32HD097807 awarded to C.B.M.).
